# Short-Term Observation of Heart Rate and Oxygen Saturation in Relation to Masticatory Muscle Activity in Temporomandibular Disorders

**DOI:** 10.3390/brainsci15040361

**Published:** 2025-03-31

**Authors:** Grzegorz Zieliński, Michał Ginszt, Marcin Wójcicki, Jacek Szkutnik, Monika Litko-Rola, Piotr Gawda

**Affiliations:** 1 Department of Sports Medicine, Medical University of Lublin, 20-093 Lublin, Poland; piotr.gawda@umlub.pl; 2 Department of Rehabilitation and Physiotherapy, Medical University of Lublin, 20-093 Lublin, Poland; michal.ginszt@umlub.pl; 3 Independent Unit of Functional Masticatory Disorders, Medical University of Lublin, 20-093 Lublin, Poland; marcin.wojcicki@umlub.pl (M.W.); jacek.szkutnik@umlub.pl (J.S.); monika.litko-rola@umlub.pl (M.L.-R.)

**Keywords:** masticatory muscles, EMG, TMDs, heart rate, oxygen saturation, temporomandibular disorders

## Abstract

(1) Background: The present study was designed to analyze the relationship between masticatory muscle activity, oxygen body saturation, and heart rate in patients with temporomandibular disorders (TMDs). (2) Methods: A total of 104 individuals with a painful form of TMDs were included in the study, consisting of 72 women and 32 men, with an average age of 24 ± 3 years. The control group included 77 individuals, comprising 44 women and 33 men, with an average age of 24 ± 3 years. An electromyographic study was conducted at rest, focusing on the temporalis (TA) and masseter muscles, alongside an oxygen saturation and heart rate analysis using a pulse oximeter. (3) Results: Analysis of the correlations between oxygen saturation and the studied muscles revealed a positive correlation between the resting activity of the TA and oxygen saturation, with a small effect size in the TMDs group. This positive correlation suggests increased muscle oxygenation is associated with higher resting activity in TMDs patients. (4) Conclusions: This study indicates that oxygen body saturation is associated with increased resting activity in the temporalis muscles of individuals with TMDs. Increased muscle tension in TMDs patients may impact the metabolism of the temporalis muscle, leading to higher energy and oxygen demands. This could be linked to hypermetabolism and an increased energy burden in these muscles at rest.

## 1. Introduction

Temporomandibular disorders (TMDs) encompass a group of conditions affecting the temporomandibular joint (TMJ), masticatory muscles, and associated anatomical structures. They manifest as pain in the jaw, face, or neck, restricted mandibular mobility, and joint sounds such as clicking or popping. TMDs are one of the most common causes of orofacial pain, significantly impacting patients’ quality of life and affecting approximately 34% of the global population [1]. The etiology of TMDs is multifactorial, involving both mechanical factors, such as excessive muscular tension, and psychological factors, including stress [2,3].

Changes in muscle tension and psychological factors are associated with modifications in the circulatory system, primarily due to the activation of the sympathetic nervous system—a component of the autonomic nervous system [4,5]. The sympathetic nervous system plays a key role in the body’s adaptive mechanisms, enhancing readiness for action by increasing heart rate, elevating blood pressure, and augmenting blood flow to muscles [6]. Over the years, numerous studies have investigated the impact of cardiovascular diseases on the function of the stomatognathic system [7,8,9,10].

For instance, Hallett et al. (1992) demonstrated that children with cardiovascular diseases, despite similar living conditions, exhibited significantly higher numbers of carious and endodontically treated teeth compared to their healthy peers. Moreover, patients with cardiovascular diseases had, on average, twice as many damaged teeth and a significantly higher prevalence of enamel defects than their siblings [7]. More recent studies by Uğurluel et al. (2022) found no significant difference in the prevalence of TMDs and oral parafunctions between children with cardiovascular diseases and the control group. However, a significant association was identified between TMDs symptoms and the presence of cardiovascular diseases, highlighting the need for further research in this area [8].

Studies on the relationship between TMDs and autonomic cardiovascular activity have shown that patients with TMDs exhibit reduced heart rate variability (HRV), particularly during nighttime, compared to healthy individuals [11]. HRV is an essential indicator of the balance between sympathetic and parasympathetic activity. In one reported case, complete resolution of temporomandibular joint pain was observed in a 28-year-old patient following cardiac surgery, suggesting potential interactions between these systems [12]. Attempts to determine whether autonomic cardiovascular parameters could serve as predictors of TMDs risk have not yielded conclusive evidence [13], but the question of circulatory alterations in individuals already affected by TMDs remains open.

A study conducted by Zegarowski demonstrated that a higher pain intensity index correlated with reduced parasympathetic activity and increased sympathetic–parasympathetic activity. The aim of this study was to determine differences in autonomic nervous system activity and masticatory muscle function in individuals experiencing TMD-related pain compared to a control group [9].

Hypothetically, TMDs may influence cardiovascular function, particularly in the context of chronic pain and muscle tension. Excessive tension in the jaw muscles may lead to sympathetic activation, resulting in increased blood pressure and heart rate. Concurrently, reduced parasympathetic activity may impair the body’s ability to recover, potentially leading to long-term health consequences [4,14].

Beyond the impact of the autonomic nervous system on the cardiovascular system, the respiratory system also plays a crucial role in masticatory muscle function, as it is regulated by the autonomic nervous system [6]. Proper blood oxygenation is essential for efficient muscle function, as oxygen is required for metabolic processes involved in energy production, such as cellular respiration. Oxygen deficiency can lead to decreased muscle efficiency, accelerated fatigue, and the accumulation of metabolic byproducts, such as lactic acid. Therefore, optimal lung ventilation, effective gas diffusion in the alveoli, and efficient oxygen transport via hemoglobin are essential for maintaining muscle function and recovery [15,16].

In the context of oxygenation and TMDs, it has been demonstrated that individuals at risk of developing TMDs may exhibit disturbances in oxygen utilization by masticatory muscles [17]. Furthermore, women with TMDs showed reduced oxygen extraction capacity in the muscles compared to healthy individuals, with symptom severity correlating with decreased O_2_ extraction [18].

As highlighted in the studies reviewed in this introduction, further research and analysis are needed to explore the interrelationship between TMDs and the cardiovascular system. Therefore, the present study was designed to analyze the relationship between masticatory muscle activity, oxygen saturation, and heart rate in TMDs patients.

Based on the aforementioned text, the research hypothesis posited that individuals with TMDs experience temporary changes in their circulatory system, including heart rate and oxygen saturation.

The study described has the following practical significance: A better understanding of the relationship between TMDs and the circulatory system may contribute to the development of more effective diagnostic and therapeutic methods that consider not only dental aspects but also systemic autonomic and cardiovascular regulations. Additionally, this knowledge may aid in the early identification of patients at increased risk of circulatory disorders, ultimately improving the quality of treatment and prevention strategies.

## 2. Materials and Methods

The study included men and women aged 20–30 years who met the following criteria: no malocclusions of Angle Class II or III, no injuries or surgeries in any part of the body within the past six months, no orthodontic treatment, no declared metallic implants regardless of location, no cancer diagnoses, no regular medication use, no any forms of TMDs (as determined by Research Diagnostic Criteria for Temporomandibular Disorders, RDC/TMD [19]) in controls, and complete dental arch support in all four quadrants. The experimental group differed from the control group by the presence of a painful form of TMDs. Muscle pain in the study group was observed in the masseter area and was present when opening the mouth, chewing food, and clenching teeth. The participants in both the control group and the TMDs group had valid occupational medicine certificates regarding their eligibility for professional work and work related to their studies. They also did not report any cardiological issues or the presence of implanted metal devices, including pacemakers.

It is also necessary to justify the choice of the age range of the study group. The age range is associated with the group most frequently exposed to TMDs. Various age ranges appear in the literature, such as 20–40 years [20] or 18–60 years [1]. The decision was made to choose the 20–30 years range because it is the starting point of these ranges, and changes in the human system at the beginning of this period can be crucial for the later clinical picture of TMDs [1,3,20].

The study was conducted in three steps. The first step involved an analysis based on inclusion and exclusion criteria. In the second step, participants underwent the RDC/TMD examination. Finally, the third step included electromyographic assessment, whole-body saturation measurement, and heart rate recording. The examiner conducting this part of the study was blinded to the participants’ group assignment (whether they belonged to the study or control group).

Electromyographic testing was conducted using the Ultium EMG system, which was compatible with myoMOTION™ (MR 3.20.62 software, Noraxon USA, Inc., Scottsdale, AZ, USA). During the procedure, participants were seated in a dental chair with their head supported on a headrest. The head, neck, and torso were maintained in a standardized position. All assessments were conducted in the hours of the morning. Initially, the skin was disinfected using 90% ethyl alcohol [21]. Electrodes with a conductive surface of 16 mm were then symmetrically applied according to the SENIAM protocol [22]. Electrode placement was preceded by a visual assessment and palpation performed by an experienced physiotherapist (the first author).

The selected muscles for the study were the temporalis muscles (TA) and masseter muscles (MM), based on the prior literature. For the analysis, the mean values from the right and left muscles examined were used [21,23]. The analysis was conducted at rest, with the participant not performing any motor tasks. The electromyograph recorded a 12 s signal, and, in accordance with the literature, the central 10 s of resting activity were analyzed [21].

**Figure 1:**
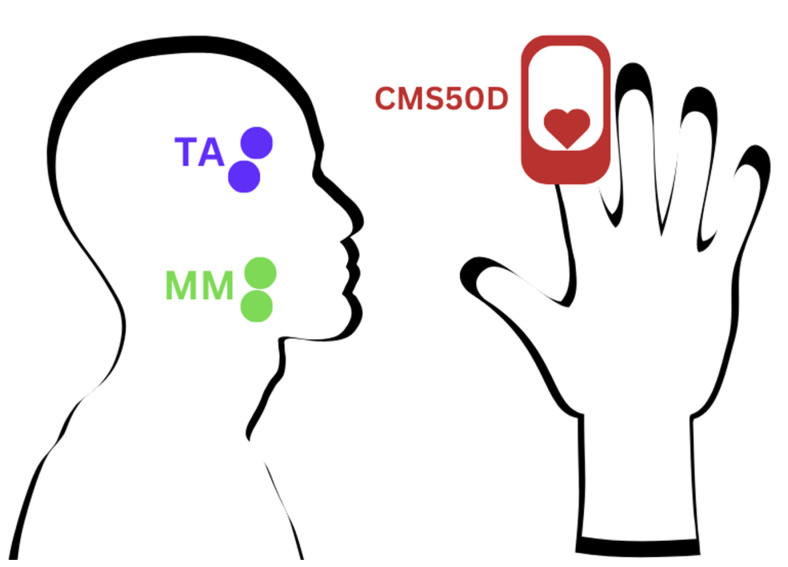
Sensor Placement. TA—electrode placement on the temporalis muscle, MM—electrode placement on the masseter muscle, CMS50D—sensor placement on the hand.

Simultaneously, the researcher monitored the SpO_2_ and heart rate readings from the Contec Pulse Oximeter CMS50D, observing the data until the signals stabilized [24,25]—Figure 1. When the researcher observed the stabilization of the results, the recording of the sEMG signal was initiated.

The device parameters were configured according to established guidelines [21,26]. The EMG signal was recorded with a sampling rate of 4000 Hz and a 24-bit internal resolution, ensuring a baseline noise level below 1 microvolt RMS. The input impedance exceeded 100 megohms, and the common mode rejection ratio was greater than 100 dB.

A hardware band-pass filter (20 Hz–500 Hz) was applied during data acquisition to remove motion artifacts and high-frequency noise, as recommended in the literature for surface EMG (sEMG) analysis. Additionally, post-processing included built-in software filters from myoMOTION™ (MR 3.20.62 software, Noraxon USA, Inc., Scottsdale, AZ, USA), following the recommended sequence for kinesiological sEMG analysis. These filters incorporated noise cleaning and signal smoothing to enhance data quality [26].

The study was approved by the local bioethics committee (BKB/947/12/2024).

Sample size calculations were performed using G*Power 3.1 software [10]. A standard alpha level of 0.05, a medium effect size of 0.5, and a power level of 0.80 were assumed [27]. Results indicated that a minimum of 51 participants per group (102 total) was required (to analyze differences between groups). For correlation analysis, assuming the same alpha level, power, and medium effect size for Pearson’s r—0.03 [27,28], the minimum sample size was 64.

Statistical analysis was conducted using Statistica™ version 13.3 (TIBCO Software Inc., Palo Alto, CA, USA). Initially, the distribution of data was examined using the Shapiro–Wilk test to determine normality. Since the data deviated from a normal distribution, nonparametric statistical methods were applied to ensure appropriate analysis. Specifically, the Mann–Whitney U-test was utilized to compare differences between groups (individuals with TMDs vs. control group), allowing for the assessment of whether TMDs significantly influence heart rate and oxygen saturation levels. Additionally, Spearman’s rho test was conducted to evaluate the strength and direction of correlations between TMDs severity and circulatory parameters. The hypotheses tested included whether there is a significant difference in heart rate and oxygen body saturation between individuals with and without TMDs. Effect sizes were interpreted according to Cohen’s guidelines, with small (0.10), medium (0.30), and large (0.50) thresholds [27,28]. Statistical significance was set at *p* ≤ 0.05. These statistical tests were chosen to align with the study’s objective of determining the extent of physiological alterations associated with TMDs while accounting for the non-normal data distribution.

## 3. Results

A total of 104 individuals with a painless form of TMDs were included in the study, comprising 72 women and 32 men, with an average age of 24 ± 3 years. The control group included 77 individuals, consisting of 44 women and 33 men, with an average age of 24 ± 3 years. Statistical analyses revealed no significant differences in gender distribution (χ^2^ = 0.79, *p* = 0.37) or age (χ^2^ = 10.39, *p* = 0.66). The differences were observed in pain-free opening: individuals with TMDs had a range of 48 mm, while healthy individuals had 50 mm. However, the results did not reach the predetermined significance threshold (Table 1).

In comparing variables such as oxygen body saturation, heart rate, and bioelectrical activity between the groups, no statistically significant differences were found (with small effect sizes). It is noteworthy that the control group exhibited lower resting muscle activity compared to the TMDs group (Table 2). Further correlation analyses were conducted within the study groups.

In the first step, an aggregate correlation was performed by combining the groups (TMDs Subjects and Healthy Subjects). None of the results from the aggregate correlations reached the predetermined level of significance (Table 3).

**Figure 2:**
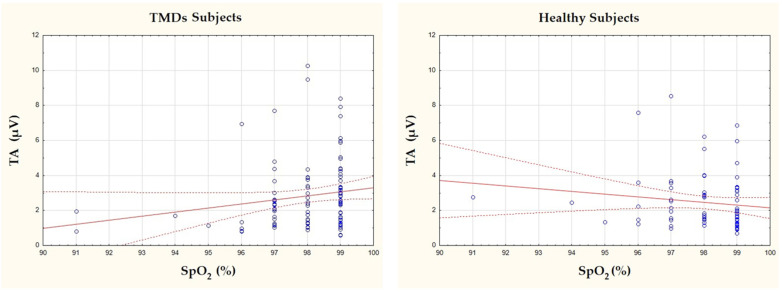
Graphical representation of the relationship between saturation and bioelectrical activity of the temporal muscle in the healthy group and the TMDs group. The solid red line represents the regression line, while the dashed line indicates the 95% confidence interval. SpO_2_—oxygen saturation; TA—the temporalis anterior.

Analyzing the correlations between oxygen body saturation and the studied muscles revealed a positive correlation between the resting activity of the temporalis muscles (TA) and body saturation, with a small effect size in the TMDs group. A positive correlation indicates that increased oxygenation of the muscle is associated with higher resting activity. However, no significant correlations were found between heart rate and bioelectrical activity (Table 4, Figure 2).

In the control group, no significant correlations were found. However, it is noteworthy that a near-significant relationship was observed between body saturation and the resting activity of the masseter muscles (MM), with a *p*-value of 0.0536 (Table 5, Figure 2).

Due to the observed positive correlation in Table 4 between whole-body saturation and the bioelectrical activity of the temporal muscle in individuals with TMDs, and the lack of such an association in the healthy group, the data were visualized using a scatter plot (Figure 2). In this figure, a rightward shift in the data cluster was observed in the TMDs group compared to the healthy group.

## 4. Discussion

The present study was designed to analyze the relationship between temporomandibular disorders, oxygen body saturation, and heart function.

The most notable finding was the correlation between saturation and the resting activity of the temporalis muscle. This result may be linked to the energy crisis observed in individuals with TMDs. Hypothetically, pain in the masticatory region reorganizes muscle activity, shifting it in favor of the temporalis muscles, which must compensate by being more active. Hence, the increased oxygen demand in TMDs patients. Increased resting activity was observed in individuals with TMDs, with higher average resting activity compared to healthy participants. However, differences between the groups did not reach statistical significance.

### 4.1. Interpretation of Finding

This could explain the observed positive correlation with oxygen saturation. It is suggested that individuals with TMDs might require more energy due to a possible increase in resting bioelectrical tension, which could contribute to heightened metabolism in these muscles. Chronic muscle tension, particularly in the temporalis muscle, has been proposed to increase oxygen demand, as tense muscles require more energy for proper function. However, the data in this study do not provide direct confirmation of this claim. This hypothesis might help explain the observed positive correlation with oxygen saturation.

Oxygen plays a critical role in maintaining muscle tone and contraction processes by supporting ATP production in cellular metabolism. Within mitochondria, oxygen is crucial for oxidative phosphorylation, where glucose and fatty acids are metabolized to produce ATP efficiently. During muscle contraction, calcium ions released from the sarcoplasmic reticulum bind to troponin, enabling the interaction of actin and myosin—key contractile proteins. ATP is required for filament sliding and the active transport of calcium ions back into the sarcoplasmic reticulum, allowing muscles to return to a resting state. Oxygen deficiency forces muscles to rely on anaerobic glycolysis, reducing ATP production and lactic acid buildup, which may contribute to fatigue and diminished muscle strength. Adequate oxygen supply supports efficient muscle function, tone maintenance, and contraction [15,16].

The lack of significant correlation in the masseter muscle may relate to its smaller size compared to the temporalis muscle. The temporalis muscle, the largest muscle in the craniofacial region and a primary muscle of mastication, requires significantly more energy to maintain adequate tension. Nevertheless, the data presented do not establish a definitive causal relationship between temporalis oxygen demand and systemic oxygenation. Previous studies, such as those by Shah et al., suggest that healthy individuals at risk for TMDs exhibit abnormalities in masseter oxygenation [17]. In this study, pulse oximetry was performed at the fingertip, which might explain some differences from Shah et al.’s findings. Given these methodological differences, direct comparisons should be interpreted with caution. Based on these results, one possible explanation is that the body responds to increased muscle tension, especially in the temporalis muscle, by activating compensatory mechanisms such as enhanced pulmonary ventilation or improved peripheral blood flow, leading to better oxygenation measured at the fingertip. This process could involve the autonomic nervous system (ANS) [11,29]. However, this remains a hypothesis and requires further empirical validation.

Ferreira et al. demonstrated that women with TMDs had reduced oxygen extraction capacity in muscles compared to healthy individuals, with greater symptom severity correlating with lower oxygen extraction [18]. This finding suggests a complex interplay between oxygen delivery and utilization rather than a straightforward increase in metabolic demand. Combining Ferreira’s findings with our results highlights a potential physiological paradox. While systemic oxygen delivery may increase, tense muscles and local circulatory disruptions could hinder efficient oxygen utilization at sites of highest demand. Thus, the relationship between systemic oxygenation and local muscle metabolism in TMDs remains unclear. These findings emphasize the complexity of TMDs pathophysiology and the need for a comprehensive research and treatment approach.

Michalek-Zrabkowska et al., conducting a systematic review on the cardiovascular implications of sleep bruxism, proposed a relationship between sleep bruxism and its cardiovascular consequences. The synthesis of evidence indicates a plausible pathophysiological mechanism linking sleep bruxism with sympathetic overactivity, which might increase cardiovascular risk in individuals with sleep bruxism [30]. This aligns with broader research on autonomic dysfunction in TMDs, highlighting systemic physiological effects beyond the masticatory system. This pattern has also been observed in the present study.

In the analysis of the results, it was observed that despite an increase in systemic SpO_2_, local tissues may experience oxygen deficiency. This could be attributed to the activation of the autonomic nervous system, particularly sympathetic stimulation [31,32]. In response to stress or hypoxia, the body increases cardiac output and redistributes blood, prioritizing their supply to key organs such as the brain and heart [33,34]. Simultaneously, vasoconstriction of peripheral vessels may limit blood flow to less critical tissues, causing a local oxygen deficit [35,36,37]. This explains why, despite an increase in SpO_2_, certain areas of the body may still suffer from oxygen deprivation.

It is worth emphasizing that the results suggest that higher oxygen saturation may be associated with increased resting activity of the temporalis muscles in patients with TMDs, indicating a greater energy and oxygen demand in these muscles. In the context of treating TMDs patients, this may suggest the need to incorporate therapies aimed at reducing muscle tension to improve metabolism and decrease the energy burden on these muscles.

### 4.2. Limitations and Future Prospects

The study did not account for all possible variables, such as stress, anxiety, physical activity, body mass index (BMI), or sleep quality, which may partially influence the physiological parameters under investigation [3,38,39,40,41]. However, every effort was made to ensure that the study group was representative, and heart rate stabilization was achieved during the examination. Additionally, we would like to note that, according to the studies, mental state, stress, and BMI did not influence the resting activity we analyzed. However, they were associated with changes during muscle function [40,41]. The analysis primarily focused on resting conditions, providing valuable insights, although it does not fully capture the body’s responses in dynamic situations, such as during chewing or in response to stress. It is worth noting, however, that including functional activity analysis (e.g., chewing or clenching) could potentially distort the obtained heart rate and oxygen saturation results. Muscle contractions may be associated with increased oxygen consumption and heightened cardiac activity. The method of analyzing only the resting activity of the masticatory muscles is widely accepted in the literature [42,43,44].

The use of a pulse oximeter to measure saturation provided valuable systemic data, although more localized measurements, such as those using Near-Infrared Spectroscopy, could have further complemented the results [45,46,47]. Nevertheless, the method used with the pulse oximeter is widely applied and accepted in medical practice. This method gained popularity during the COVID-19 pandemic [24,25,48]. In terms of muscles, pulse oximeters are primarily used in polysomnographic studies analyzing the phenomenon of bruxism, which is a widely used practice [49,50,51,52]. It is worth noting that there may be discrepancies between the analysis of heart rate and saturation parameters when using a fingertip sensor compared to a sensor placed on the forehead. In this context, it is relevant to mention the findings of Wilson et al., who observed that those using fingertip sensors should consider that oxygen saturation may be up to 5% higher when using a sensor on the forehead [53]. It is important to note that alternative methods for analyzing heart rate and pulse closer to the craniofacial area could interfere with the operation of the electromyographic device.

The authors note a possible association between increased resting activity of the temporalis muscle and higher oxygen demand, emphasizing that this is a correlation that requires further investigation. Potential alternative mechanisms, such as inflammation or sympathetic nervous system hyperactivity, may also be relevant and could be an interesting direction for future analyses [32].

The cross-sectional design restricts the ability to determine causality, making it difficult to establish whether increased TA activity influences SpO_2_ levels or the other way around. Therefore, it is essential to emphasize the need for longitudinal or interventional studies to clarify these causal relationships.

Future scientific research should focus on further exploring the relationship between TMDs and cardiovascular system function. It will be essential to determine the mechanisms through which TMDs may influence circulatory parameters, including heart rate variability and blood pressure.

Further studies should also consider the impact of chronic pain and excessive muscle tension on autonomic nervous system activity, particularly regarding the balance between the sympathetic and parasympathetic systems. Another crucial research area is the role of oxygenation in patients with TMDs, especially in the context of oxygen transport efficiency and its utilization by the masticatory muscles.

An important aspect of future research is the assessment of masticatory muscle endurance in patients with TMDs, particularly in the context of chronic muscle hyperactivity, which may lead to impaired regeneration and increased fatigue [54].

Additionally, future analyses should include potential differences in the correlations between TMDs and circulatory parameters across different population groups, considering factors such as age, sex, and the presence of comorbidities. Another key research direction is the identification of biomarkers indicating the risk of TMDs in relation to cardiovascular parameters, which could contribute to earlier diagnosis and more effective therapeutic strategies.

Ultimately, long-term prospective studies are necessary to determine whether temporomandibular disorders increase the risk of developing cardiovascular diseases in the future, which would have significant clinical and preventive implications.

## 5. Conclusions

This study indicates that oxygen saturation is associated with increased resting activity in the temporalis muscles within individuals with painful forms of TMDs. Increased muscle tension in TMDs patients may impact the metabolism of the temporalis muscle, leading to higher energy and oxygen demands, which may be linked to hypermetabolism and increased energy burden in these muscles at rest.

## Figures and Tables

**Table 1:** Presentation of mandibular range of motion in the groups.

	TMDs Subjects		Healthy Subjects		Z	*p*
	Mean	SD	Mean	SD		
Pain-free opening	48	8	50	6	0.59	0.55
Maximum unassisted opening	51	6	50	6	−0.58	0.56
Maximum assisted opening	54	6	53	6	−1.36	0.17
Mandibular movement to the right	10	2	9	2	−0.89	0.38
Mandibular movement to the left	11	2	10	2	−2.99	0.00
Protrusion	9	2	9	2	−0.83	0.41

**Table 2:** Comparison of saturation, heart rate, and TA and MM bioelectrical activity between groups.

	TMDs Subjects		Healthy Subjects		Z	*p*	ES
	Mean	SD	Mean	SD			
SpO_2_ (%)	97.97	1.44	98.03	1.70	−0.66	0.51	0.05
HR (bpm)	73.35	13.48	73.09	17.68	0.96	0.34	0.07
TA (μV)	2.83	1.95	2.45	1.59	1.17	0.24	0.09
MM (μV)	2.39	1.42	2.23	1.07	−0.01	0.99	0.00

SpO_2_—oxygen saturation; HR—heart rate; bpm—beats per minute; TA—the temporalis anterior; MM—the superficial part of the masseter muscle; μV—microvolt; ES—effect size.

**Table 3:** Correlation analysis between saturation, heart rate, and studied muscles in individuals.

SpO_2_ (%)			Mean	SD	R	t(N-2)	*p*
Mean	SD						
97.99	1.55	TA (μV)	2.67	1.81	0.09	1.27	0.21
		MM (μV)	2.32	1.28	0.01	0.10	0.92
**HR (bpm)**							
**Mean**	**SD**						
73.24	15.36	TA (μV)	2.67	1.81	−0.01	−0.18	0.86
		MM (μV)	2.32	1.28	−0.03	−0.35	0.73

SpO_2_—oxygen saturation; HR—heart rate; bpm—beats per minute; TA—the temporalis anterior; MM—the superficial part of the masseter muscle; μV—microvolt.

**Table 4:** Correlation analysis between saturation, heart rate, and studied muscles in individuals with TMDs.

	R	t(N-2)	*p*
SpO_2_ (%)			
TA (μV)	0.20	2.06	0.04 *
MM (μV)	0.15	1.47	0.14
HR (bpm)			
TA (μV)	−0.13	−1.36	0.18
MM (μV)	0.04	0.44	0.66

SpO_2_—oxygen saturation; HR—heart rate; bpm—beats per minute; TA—the temporalis anterior; MM—the superficial part of the masseter muscle; μV—microvolt; * Significant difference.

**Table 5:** Correlation analysis between saturation, heart rate, and studied muscles in healthy individuals.

	R	t(N-2)	*p*
SpO_2_ (%)			
TA (μV)	−0.04	−0.34	0.73
MM (μV)	−0.22	−1.96	0.05
HR (bpm)			
TA (μV)	0.13	1.11	0.27
MM (μV)	−0.14	−1.23	0.22

SpO_2_—oxygen saturation; HR—heart rate; bpm—beats per minute; TA—the temporalis anterior; MM—the superficial part of the masseter muscle; μV—microvolt.

## Data Availability

The raw data supporting the conclusions of this article will be made available by the authors on request.
